# Manifestation and Associated Factors of Pregnancy-Related Worries in Expectant Fathers

**DOI:** 10.3389/fpsyt.2020.575845

**Published:** 2020-12-11

**Authors:** Ariane Göbel, Petra Arck, Kurt Hecher, Michael Schulte-Markwort, Anke Diemert, Susanne Mudra

**Affiliations:** ^1^Department of Child and Adolescent Psychiatry, Psychotherapy and Psychosomatics, University Medical Center Hamburg-Eppendorf, Hamburg, Germany; ^2^Department of Obstetrics and Fetal Medicine, University Medical Center Hamburg-Eppendorf, Hamburg, Germany

**Keywords:** pregnancy-related worries, fatherhood, paternal, prenatal mental health, prenatal care, pregnancy, Cambridge Worry Scale

## Abstract

**Background:** During the last decades, fathers have increasingly participated in prenatal care, birth preparation classes, and childbirth. However, comparably little is known about the prenatal emotional well-being of fathers, particularly content and extent of broader paternal concerns that may arise during pregnancy beyond those focusing on childbirth. Thus, the aims of this study were to investigate the manifestation of paternal pregnancy-related worries in a population-based sample and to identify relevant associated factors.

**Materials and Methods:** As part of a longitudinal pregnancy cohort at the University Medical Center Hamburg-Eppendorf, Germany, *N* = 129 expectant fathers were assessed once during pregnancy. Pregnancy-related worries centering around medical procedures, childbirth, health of the baby, as well as socioeconomic aspects were assessed with the Cambridge Worry Scale (CWS). Additionally, paternal socioeconomic background and maternal obstetrical history, symptoms of generalized anxiety and depression, and level of hostility were investigated, as well as perceived social support. The cross-sectional data were analyzed based on multiple regression analyses.

**Results:** The level of reported worries was overall low. Some fathers reported major worries for individual aspects like the health of a significant other (10.9%) and the baby (10.1%), as well as the current financial (6.2%) and employment situation (8.5%). Pregnancy-related worries were negatively associated with household income and positively associated with anxious and depressive symptoms and low perceived social support. Associations varied for specific pregnancy-related worries.

**Limitations:** Due to the cross-sectional data examined in this study, a causal interpretation of the results is not possible. The sample was rather homogeneous regarding its socioeconomic background. More research needs to be done in larger, more heterogeneous samples.

**Conclusion:** Though overall worries were rather low in this sample, specific major worries could be identified. Hence, addressing those fathers reporting major worries regarding specific aspects already in prenatal care might support their psychosocial adjustment. Fathers with little income, those with elevated levels of general anxious and depressive symptoms, and those with less social support reported higher pregnancy-related worries. Our results indicate the relevance of concerns beyond health- and birth-related aspects that could be relevant for fathers. Measurements developed specifically for expectant fathers are needed to properly capture their perspective already during pregnancy.

## Introduction

Even though most parents perceive the transition to parenthood as a positive experience with excitement and joy, often negative feelings and insecurity are a substantial part of becoming a mother (1) or father (2, 3). Hence, some parents develop high levels of anxiety or suffer from pregnancy-related anxiety and worry with potential negative consequences of maternal pregnancy-related worries for the health and development of the baby (1, 4–6). For a long time, research on perinatal emotional adjustment has focused mainly on mothers, despite a societal shift in the perspective on fatherhood toward fathers being a more active caregiver for the child (7–10). Supporting this change in the understanding of paternal roles, previous studies indicate that active, supportive paternal caretaking is positively associated with the quality of the father–child relationship and child's socioemotional (11–15) and cognitive development (16–18). Paternal involvement might further promote perinatal mental health of the mother (19) and birth-related outcomes for the infant (20, 21) and function as a buffer against potentially negative effects of maternal depression or parenting stress on the family (19, 22, 23). Research indicates associations of higher paternal engagement and more optimal quality of co-parenting with less maternal parenting distress and a positive outcome regarding child's socio-emotional development also in families where parents were not or are no longer in a romantic relationship and families with socioeconomic risk factors (24–27). Qualitative studies indicate that fathers perceive an active involvement in perinatal care and birth as central for their transition to fatherhood (28). However, even though fathers report interest in participating in prenatal care, birth preparation classes and delivery itself, they might also feel that the focus is on their pregnant partner and the baby, giving themselves less space to find their own role, and to express distress or concerns in the context of pregnancy and childbirth (29–32). They might further feel the need to hold back potential negative feelings to not worry their pregnant partner (33) or because of a socialized stereotypical understanding of the expression of anxieties as a sign of male weakness (34). Intense insecurity and pregnancy-related concerns that are not acknowledged might lead to a withdrawal of fathers from the prenatal care context or delivery and from their partner and child (35, 36) and by this hinder the development of a parental identity and the father–child relationship. Thus, it is important to better understand the manifestation of paternal concerns related to pregnancy and childbirth and their associated factors, which has rarely been focus in the literature so far.

### Pregnancy-Related Worries

During the last decade, the focus on mental health in the peripartum period has gained increased attention. Research in mothers showed that prenatal emotional distress is associated with negative consequences for the health of mother and baby and needs to be identified and addressed as early as possible (4, 6, 37–40). In this context, pregnancy-related anxiety has been identified as a distinctive entity of unique and high relevance for outcomes in mother and child, like birth and pregnancy complications (41–44), infant's temperament (5, 45), child's subsequent cognitive and socioemotional development (45–48), as well as maternal postpartum adjustment, mood, and parenting stress (42, 49–52).

In the literature, a variety of conceptualizations of pregnancy-related anxiety exists (53–58). In a qualitative analysis, Bayrampour and colleagues proposed a definition of the concept as “nervousness and fear about the baby's health, the mother's health and appearance, experience with the health care system, social and financial issues in the context of pregnancy, childbirth, and parenting that are accompanied by excessive worry and somatic symptoms” [(58) p. 121]. Only few studies have examined pregnancy-related anxiety or worries in fathers or investigated the psychometric properties of available instruments for the use in men (59). One reason for this might be that many questionnaires on pregnancy-related anxiety focus, besides aspects like childbirth or the health of mother and baby, mainly on the maternal perspective or on medical or bodily experiences of being pregnant (55, 60, 61), which are difficult to directly transfer to the father's perspective.

Previous studies on pregnancy-related concerns in fathers have moreover often focused specifically on fears related to childbirth. Hanson et al. (34) described in a literature review on paternal childbirth-related fear that, across the included studies, the most prevalent concerns center around the well-being or potential death of the partner during labor, followed by fear for the health of the unborn baby. Lindgren et al. (62) reported that strong birth- and health-related worries assessed with the Cambridge Worry Scale (CWS) were lower in Swedish fathers than in mothers and decreased in both parents from expecting to receiving results of the first trimester screening. In a Turkish sample, 54.2% of expectant fathers compared to 82.6% of mothers reported high levels of childbirth-related anxiety as assessed with the Fear of Birth Scale (63). In a study by Eriksson et al. (33, 64), 59% of their Swedish sample reported mild to moderate levels and 13% intense levels of childbirth-related fear assessed with a self-developed set of questions. The group of highly anxious fathers reported intense mental preoccupation with these fears, a state of higher vigilance, and bodily reactions (33). Bergström et al. (65) found that Swedish fathers with intense levels of childbirth-related fear (10.9%) assessed with an adapted version of the Wijma Delivery Experience Questionnaire had an increased risk for feeling unprepared for childbirth and for experiencing childbirth as frightening. These anxieties might also influence the experience of pregnancy and involvement related to perinatal care. In a study by Hildingsson et al. (35), Swedish fathers with high childbirth-related fear (13.6%) assessed with the Fear of Birth Scale more often perceived difficulties during pregnancy and the forthcoming birth, and highly anxious first-time fathers less often attended antenatal education classes than those without fear. Further, highly anxious fathers reported lower prenatal mental and physical health and experienced higher parenting stress 1 year postpartum. In Serçekuş et al. (63) study, highly anxious fathers preferred a cesarean section instead of a vaginal birth. Greer (66) reported in a qualitative analysis that men in their sample from Northern Ireland perceived a medically supported (e.g., epidural anesthesia) or managed (planned caesarian section) birth as safer, easier to control, and better to cope with. In a German longitudinal investigation, prenatal birth concerns (assessed with a self-developed questionnaire) were identified as a distinct predictor for depressive symptoms within the first 6 weeks postpartum (67). Finally, in Norwegian fathers, pregnancy-related worries predicted the perception of the infant's behavior at 6 and 12 months as more difficult (68, 69). These results and their potential indication for birth-related decisions, paternal involvement, and postnatal adjustment underline the relevance of better understanding and addressing paternal concerns in the perinatal period.

Since having a child affects many different aspects in one's life, concerns for expectant fathers might also be associated with domains like caregiving competence and important relationships, as well as the living and financial situation. Previous studies frequently described that fathers felt unprepared or even inadequate for their role as a father (34, 70, 71). A broader understanding of the concept of pregnancy-related worries (58) might not only address the maternal but also the paternal perspective more sufficiently.

However, few studies have explicitly investigated a broader range of paternal pregnancy-related worries. Forsyth et al. (72) found in an Australian sample of expectant fathers that not being able to provide for the family was among the strongest worries, right after being concerned about the pregnant partner experiencing pain and their child being born handicapped. Other worries were related to not being a good enough father, insufficiently supporting their partner, the quality of the partner relationship, as well as not being involved enough or not being present during birth. These results are comparable to the results by Biehle and Mickelson (73) in an American sample, further reporting sex differences, with fathers being more likely to worry about financial and work-related aspects than their pregnant partner. On the other hand, in a German study by Kannenberg et al. (74), concerns regarding the economic situation and parenting were in both expectant mothers and fathers less relevant than those regarding the health and development of the baby and problems during delivery. Due to the few investigations with a broader perspective on pregnancy-related concerns, more research on the relevance of specific concerns in fathers is needed.

Further, the assessment of fear of childbirth, as one specific aspect of pregnancy-related anxiety, often focuses on the identification of expectant parents with high or even pathologic levels of fear. In contrast, given the little research on different pregnancy-related worries in fathers and the lack of valid father-specific instruments thus far, the investigation of a wider concept and different levels of prenatal worries are needed to contribute to the understanding of the paternal perspective before further differentiation. An assessment of the content and extent of worries closely related to pregnancy as well as general worries in expectant parents during pregnancy that are non-pathologic, but might still substantially increase distress, is important (75, 76). Hence, to fully understand relevant paternal concerns in the perinatal period, it is of high interest to investigate different pregnancy-related worries, beyond those focusing on childbirth. The CWS (75) is one of the few existing questionnaire-based instruments (61, 77–79) that allow the quantitative assessment of a broader range of pregnancy-related worries and further have the potential to be adapted to the paternal perspective as well (59, 65). The CWS has been translated to Spanish, Greek, Swedish, Turkish, Portuguese, Dutch, and Farsi for use in maternal samples (80–84). To the knowledge of the authors, the CWS has in expectant fathers so far only been used in Swedish samples (62, 65). Finally, next to assessing extent and content of pregnancy-related concerns, investigating contextual and personal influencing factors is of further interest to identify those men in need for special support.

### Associations With Sociodemographic Factors

Contextual aspects of the socioeconomic background might especially be relevant when having a child. Lower income has been associated with higher levels of prenatal general anxiety in men (3) and childbirth-related fear in women (49, 85). In contrast, Serçekuş et al. (63) found higher child-birth related concerns in couples with higher socioeconomic status, which the authors explained with easier access to internet-based and by this potentially more ambiguous or incorrect information that might increase pregnancy-related worries. Generally, since in many families paid employment is distributed between both partners (9, 86), the perspective of being the main provider for the family, particularly during maternity leave, and being faced with the expenses going along with a growing family could increase the perceived economic pressure in some fathers. Paternal concerns might thus also center around changes in living or employment conditions (72, 73, 87), especially in families with lower income. Previous research has further indicated that paternal age might have an effect on the experience and concerns on parenthood. While some studies reported that fathers with advanced age (≥35 years) reported higher levels of fear of childbirth than younger fathers (88), or did not show a significant association with age (35, 89), others indicate that younger expectant fathers were at higher risk for developing pregnancy-related worries (63, 74).

### Associations With Obstetric Factors

Studies on perinatal adjustment indicate that those fathers expecting the first child might feel a higher level of insecurity during pregnancy and in preparation of childbirth than those who already have children (3). In line with findings in primiparous mothers (51), this might also lead to higher pregnancy-related worries in fathers (35, 63). The obstetric history of the pregnant partner might further influence paternal concerns. Miscarriages are not uncommon and affect in early pregnancy up to one out of five women (90), with a negative influence on psychological well-being also in fathers (91, 92). Previous qualitative and quantitative studies indicate that fathers might feel a higher sense of risk during a subsequent pregnancy after perinatal loss and also report higher pregnancy-related worries (93–96). A history of previous miscarriages might increase current pregnancy-related worries centering especially around medical procedures, the progress of pregnancy, or the health of mother and child.

### Associations With Psychosocial Factors

Pregnancy has been identified as the most stressful period during the transition to fatherhood (97, 98), which might be due to the major transformations of many aspects in the parent's life, personal roles, and identities (87, 99, 100). Even though pregnancy-related worries have been identified as entity distinct from general forms of worry and anxiety in pregnant women (4), previous studies in expectant mothers (101–104) showed an association of prenatal perceived distress, anxiety, and depression with elevated levels of pregnancy-related worries. Positive associations of prenatal levels of anxiety and perinatal depression have also been reported in men (59). Due to the few studies on pregnancy-related anxiety in fathers, the role of psychosocial distress, such as the association between symptoms of anxiety disorders and depression and the extent or form of different pregnancy-related worries in particular, still needs to be examined. Negative emotionality in the prenatal period has further been associated with higher irritability or anger and gender role stress in both women and men (105, 106). However, expression of distress might differ in women and men (107). In particular, men might be socialized based on traditional gender norms in a way that discourages them from reporting impaired mood or negative affect. These men, fearful of expressing depressive or anxious emotions, might express distress in rather externalizing symptoms like substance abuse, avoidance, or aggressive or hostile behavior (36). Hence, besides generalized anxiety and depression, the examination of hostility as a specific form of worrisome paternal distress in the context of pregnancy-related concerns might give a deeper insight into the prenatal paternal perspective.

On the other hand, perceived social support has been identified as one important protective factor in relation to mental health, also in the context of pregnancy and paternal distress (e.g., (3, 59, 108). It has previously been described that compared to their partner, many fathers less often actively seek social support during the demanding peripartum period, but rely in their emotional needs on the support from their partner (109). However, the availability of a close social support network and receiving emotional support from peers, family, or friends might function as a buffer against distress in the prenatal period also for expectant fathers, with positive consequences for their emotional well-being (97).

### Aims of This Study

The literature listed above highlights the relevance of investigating pregnancy-related worries also in men. It also shows that most previous research has focused on paternal childbirth-related concerns. Given the few investigations on prenatal anxiety and worry in fathers thus far, the first aim of our study was to assess the relevance and broader range of worry that may arise in expectant fathers. To the knowledge of the authors, the CWS has not been used to assess pregnancy-related worries in a German paternal sample before. The second aim was to investigate the association of specific sociodemographic, obstetric, and psychosocial factors with pregnancy-related worries when included into one statistical model. Based on previous results in the literature, we expected that younger age, lower income, higher psychological distress (in the form of higher levels of generalized anxiety, depression, and hostility), and less perceived social support to be associated with higher levels of pregnancy-related concerns.

## Materials and Methods

### Study Design

The data for this cross-sectional analysis were derived from a collaboration between two related ongoing population-based prospective pregnancy cohorts, carried out at the University Medical Center Hamburg-Eppendorf, Germany [PRINCE—“Prenatal Identification of Children's Health,” for details, see (110), and PAULINE—“Prenatal Anxiety and Infant Early Emotional Development,” see (104)]. Data were collected via self-report questionnaires in the second to third trimester of pregnancy. Participants signed informed consent forms. The study protocols were approved by the ethics committee of the Hamburg Chamber of Physicians (PV3694, PV5574).

### Study Sample and Procedure

To assess a population-based low-risk sample, participants were recruited between 2014 and 2018 when accompanying their pregnant partner to a study appointment. Of the 284 women reporting being in a relationship, in 129 cases, their partner joined them to one of their appointments and agreed to participate (45.42%). Detailed information on why the partner did not attend the appointment or agreed to participate was not available. Couples expecting a singleton child were included in the study. Pregnancies with chronic infections, substance abuse, or conception after assisted reproductive technologies were excluded, as were participants younger than the legal age of 18 and with poor understanding of German. Participants were asked to fill out a set of questionnaires independently from their pregnant partner.

### Variables and Instruments

#### Pregnancy-Specific Worries

In contrast to other prenatal instruments, the CWS (75) enables the quantitative assessment of a broader variety of worries potentially relevant during pregnancy beyond the focus on childbirth and health-related issues. In a German sample of pregnant women, the subscales *socioeconomic and relationships* (e.g., your housing, money problems, employment, relationship with partner, relationship with family and friends), *socio-medical* (e.g., going to hospital, internal examinations, giving birth, coping with the new baby), *health of the baby* (e.g., the possibility of something being wrong with the baby, possibility of miscarriage), and *health of oneself/someone close* were identified (76). Item scores range from 0 (“no worry”) to 5 (“major worry”). In line with previous research, a mean scale score of ≤3 was considered “less than major worry” and ≥4 was considered as “major worry” (62, 111). The CWS allows for modifying the combination of items for use specified to the research question. Therefore, we excluded two items that were not relevant for any of the subscales and typically rather focus on maternal concerns (whether partner would attend birth, giving up work). In a Swedish paternal sample, good reliability for the total score was reported [Cronbach's alpha = 0.81, (112)]. In our sample, scale reliability of the total score was comparable (α = 0.83). For the main analysis, the mean scale score of the total CWS was used as outcome variable.

#### Symptoms of Generalized Anxiety

The seven-item Generalized Anxiety Disorder Scale [GAD-7, (113)], which is a one-dimensional screening instrument, was used to assess symptoms of Generalized Anxiety Disorder (GAD) within the last 2 weeks (e.g., “feeling nervous, anxious or on edge”). The GAD-7 is a frequently used questionnaire with confirmed validity and reliability (114). Items are rated on a four-point scale, and the total score ranges from 0 to 21. A score of ≥10 moderate and a score of ≥15 severe levels of GAD (113). In the current study, scale reliability was good (α = 0.83).

#### Depressive Symptoms

The 10-item Edinburgh Postnatal Depression Scale [EPDS; (115)] was used for the assessment of depressive symptoms within the last week (e.g., “I have felt sad or miserable”). Originally developed for the assessment of maternal depressive symptoms in the postnatal period, the psychometric properties of the EPDS have been confirmed in populations in the pre- and postnatal period, as well as independent of gender (116, 117). Items are rated on a four-point scale, and scale scores range from 0 to 30. For the EPDS, different cutoff scores have been identified related to gender. Based on the previous literature, a score ≥9 was considered indicative of minor and major depressive symptoms (67, 116). In the current study, scale reliability was good (α = 0.82).

#### Hostility

General hostility was assessed with the hostility subscale of the Brief Symptom Inventory [BSI; (118)], assessing instances of hostile thoughts, annoyance, argumentative tendencies or uncontrollable anger outbursts [e.g., feeling easily annoyed and irritated; frequently getting into arguments; (118)]. The subscale consists of five items, answered on a four-point scale. The mean scale scores, ranging from 0 to 4, were calculated. Higher scores indicate higher levels of hostility. In the current study, scale reliability was acceptable (α = 0.71).

#### Perceived Social Support

Perceived social support was assessed with the Berlin Social Support Scale [BSSS; (119)]. Items are rated on a scale from 1 to 4, with a higher score indicating higher perceived social support. Originally designed for and validated in cancer surgery patients, the BSSS is used beyond this scope, including prenatal research (120, 121). Eight items assess the amount of perceived emotional (e.g., “There is always someone there for me when I need comforting”) and instrumental (e.g., “There are people who offer me help when I need it”) social support. Higher mean scale scores indicate higher satisfaction with perceived social support. In the current study, scale reliability was good (α = 0.87).

#### Sociodemographic Information and Obstetric Data

Sociodemographic information regarding paternal age, education level, and household income were also assessed. Information regarding previous children and previous miscarriages were collected from the participating women at study entry.

### Statistical Analysis

Descriptive statistics were used to investigate the distribution of CWS item and scale scores, as well as of the included potentially associated variables. Bivariate associations of pregnancy-related worries with the remaining variables were investigated using Pearson correlations. To analyze the question under research, multiple linear regression analyses were conducted. Due to its variation within this sample and previously reported changes of paternal pregnancy-related worries across pregnancy (74, 89, 122), gestational age at assessment was entered as potential control variable. Next, the contextual variables paternal age and household income, the obstetric variables previous children (dummy coded no = 0, yes = 1) and miscarriages (dummy coded no = 0, yes = 1), as well as the psychosocial variables general anxious and depressive symptoms, hostility, and perceived social support were included. Associations were considered significant at *p* ≤ 0.05, two-tailed. Missing data points in the variables investigated as associated factors were replaced using expectation-maximization imputation. Statistical assumptions for multiple regression analyses were fulfilled. For all analyses, IBM SPSS, Version 22 (123) was used.

## Results

### Sample Characteristics

Characteristics of the sample are listed in Table 1. The assessment point ranged from the beginning of the second to the end of the third pregnancy trimester. Overall, the sample was rather homogeneous regarding the socioeconomic background. Two-thirds of the sample had a high school to university degree, which was average to high compared to German statistics for men in this age range (124). Household income can be considered comparable to the average household income for couples with one or more children in Germany (125), with 2.4% having an income below 1,000€, 4.7% between 1,001 and 2,000€, 35.7% between 2,001 and 4,000€, and 51.2% above 4,000€ (6% did not provide information on their income). Mean scores in the psychosocial questionnaires are listed in Table 2 and indicate a variance in scores from low to substantial anxious and depressive symptom levels. Mean scores of the GAD-7 are above the norm values for the general German population, but below those for samples in primary care (114, 126), with 6.1% reporting moderate and 1.6% reporting severe levels of anxiety. Mean scores of depressive symptoms are comparable to another study using the EPDS prenatally in a German paternal sample (67), even though the rate of 13.5% above the cutoff was higher in the current sample. Hostility levels were low to moderate and slightly higher than the norm values reported for the validation sample by Derogatis and Spencer (118), but lower than the values reported for German men after psychotherapeutic treatment (127). Level of perceived social support ranged from average to high, with all scores above the mean value and 50% of the sample reporting high social support.

**Table 1:** Baseline characteristics of the cohort (*N* = 129).

Paternal age at assessment, in years; *M* (*SD*)	35.1	(4.47)
Range		24 to 49
Gestational age at assessment, in weeks; *M* (*SD*)	27.68	(9.83)
Range		12 to 41
Paternal education, *n* (%)		
Main or middle school	40	(31.0)
High school graduation	49	(38.0)
University degree	38	(29.5)
No information provided	2	(1.6)
Number of previous children^a^, *n* (%)		
None	79	(61.2)
One or more children	48	(37.3)
No information provided	2	(1.6)
Miscarriages of partner^a^		
No	98	(76.0)
Yes	23	(17.8)
No information provided	8	(6.2)

a *Information provided by the pregnant partner at study entry*.

**Table 2:** Descriptive statistics of the paternal psychosocial variables (*N* = 129).

	***M***	***SD***	**Range**
Generalized anxiety symptoms (GAD-7)	4.02	3.41	0 to 18
Depressive symptoms (EPDS)	4.14	3.77	0 to 18
Hostility (BSI)	0.54	0.51	0.0 to 2.0
Perceived social support (BSSS)	3.76	0.32	2.6 to 4.0

*GAD-7, Seven-item Generalized Anxiety Disorder Scale; EPDS, Edinburgh Postnatal Depression Scale; BSI, Brief Symptom Inventory; BSSS, Berlin Social Support Scale*.

### Characteristics of Pregnancy-Related Worry Items

Descriptive statistics of the individual items are listed in Table 3. Overall, the mean score of pregnancy-related worries was low, with only 0.8% of the sample reaching a total score of 3, indicating less than major worry (62, 111). On item level, mean scores were below average, ranging from *M* = 1.77 to 0.16. However, specific concerns were reported as major worries, as indicated by a score of 4 or higher. The strongest concerns in this sample centered around the health of a significant other with the highest mean score (*M* = 1.77) and 10.9% reporting it as a major worry, as well as the health of the baby (*M* = 1.66), which was a major worry for 10.1%. Next, the financial situation (*M* = 1.50) was reported as a major concern by 6.2%, and employment problems (*M* = 1.40) by 8.5% of the sample. Childbirth itself was rated by 3.1% as a major concern (*M* = 1.39), as were concerns about the own health (*M* = 1.31). For coping with the baby (*M* = 1.05) and the possibility of a miscarriage (*M* = 1.05), comparable mean scores were reported; however, the latter was, for more men, a major concern (5.5%), compared to coping with the baby (2.4%). Three percent of men were concerned about the possibility of a preterm birth. The housing situation (*M* = 0.97) and relationships with family/friends (*M* = 0.84) were major concerns for 3.9% and 3.1% of the sample. Internal examinations were overall scored even lower (*M* = 0.74) and were a major concern for 3.2%. The relationship with the partner (*M* = 0.62) and going to the hospital (*M* = 0.69) were for more than half of the sample no worry at all. Conflicts with the law were no relevant concerns in this sample (*M* = 0.16).

**Table 3:** CWS item characteristics.

	**Distribution of item responses (%)**						**Mean score**	
**Individual items**	**0**	**1**	**2**	**3**	**4**	**5**	***M***	***SD***
Health of someone close	20.9	21.7	28.7	17.8	10.1	0.8	1.77	(1.29)
Possibility of sth. wrong with the baby	19.4	27.9	31.8	10.9	8.5	1.6	1.66	(1.24)
Financial problems	19.4	35.7	26.4	11.6	5.4	0.8	1.50	(1.14)
Employment problems	24.8	38.8	16.3	11.6	8.5	0.0	1.40	(1.22)
Childbirth itself	24.0	28.7	34.9	9.3	3.1	0.0	1.39	(1.05)
Your own health	23.3	37.2	28.7	7.8	2.3	0.8	1.31	(1.04)
Coping with the new baby	39.5	29.5	20.2	8.5	1.6	0.8	1.05	(1.10)
Possibility of a miscarriage	36.4	38.0	15.5	4.7	4.7	0.8	1.05	(1.12)
Possibility of preterm birth	36.4	37.2	18.6	3.9	3.1	0.0	0.99	(1.00)
Housing situation	44.2	28.7	17.1	6.2	3.9	0.0	0.97	(1.10)
Your relationship with your family/friends	50.4	27.1	14.7	4.7	2.3	0.8	0.84	(1.07)
Internal examinations	52.7	31.0	10.1	3.1	1.6	1.6	0.74	(1.04)
Your relationship with your partner	58.9	27.1	8.5	3.9	1.6	0.0	0.62	(0.91)
Going to hospital	53.5	27.9	14.7	3.9	0.0	0.0	0.69	(0.86)
Conflict with the law	88.4	7.8	3.9	0.0	0.0	0.0	0.16	(0.46)
**Total scale and subscale scores**	**Distribution of mean scale scores (%)**						**Mean score**	
	**<1**	**≥1**	**≥2**	**≥3**	**≥4**	**5**	***M***	***SD***
CWS total score	46.5	47.2	5.5	0.8	0.0	0.0	1.07	0.57

### Bivariate Correlations

Correlations of the CWS total score with the included variables are listed in Table 4. Regarding the sociodemographic variables, small- to medium-sized negative correlations were found for the CWS total score with paternal age and household income. The CWS total score was further positively associated with symptoms of general anxiety, depression, and hostility, with small to moderately sized correlations. Finally, a negative, small to moderately sized association of the CWS total score with perceived social support was found. Thus, on the bivariate level, younger men, those with lower household income, those with higher levels of general anxiety and depression, and those with lower perceived social support also reported higher levels of pregnancy-related worries. Also, a negative trend of the CWS total score with previous miscarriages was observed.

**Table 4:** Bivariate associations between pregnancy-related worries and included variables.

		**1**	**2**	**3**	**4**	**5**	**6**	**7**	**8**	**9**	**10**
1	Overall pregnancy-related worries (CWS)	-	−0.13	−0.22^*^	−0.23^*^	−0.07	−0.16^+^	0.38^**^	0.39^**^	0.22^*^	−0.23^**^
2	Gestational age		−	0.11	0.03	−0.04	−0.11	0.02	0.02	0.18^*^	−0.05
3	Paternal age			−	0.23^**^	0.17	0.16	−0.02	−0.07	−0.09	−0.13
4	Household income				−	−0.13	0.02	−0.02	−0.01	0.12	0.02
5	Previous children (no/yes)					−	0.23^**^	0.01	0.07	0.17	−0.12
6	Miscarriages (no/yes)						−	−0.03^*^	−0.20	−0.16	0.20^*^
7	Generalized anxiety symptoms (GAD-7)							−	0.68^**^	0.57^**^	−0.09
8	Depressive symptoms (EPDS)								−	0.54^**^	−0.13
9	Hostility (BSI)									−	−0.21^*^
10	Perceived social support (BSSS)										−

** *p < 0.01*,

* *p < 0.05*;

+ *p = 0.05*.

*CWS, Cambridge Worry Scale; GAD-7, seven-item Generalized Anxiety Disorder Scale; EPDS, Edinburgh Postnatal Depression Scale; BSI, Brief Symptom Inventory; BSSS, Berlin Social Support Scale*.

### Regression Analyses

To investigate the amount of explained variance of the included predictor variables in pregnancy-related worries, multiple regression analyses were calculated. Results are listed in Table 5.

**Table 5:** Prediction of overall pregnancy-related worries.

**Variable**	***B***	**SE *B***	**β**	***p***
Constant	54.79	10.35		0.000
Gestational age	−0.11	0.07	−0.13	0.107
Paternal age	−0.25	0.15	−0.14	0.098
Household income	−**0.98**	**0.36**	−**0.22**	**0.007**
Previous children (no/yes)	−2.24	1.46	−0.13	0.127
Miscarriages (no/yes)	−0.86	1.85	−0.04	0.642
Generalized anxiety (GAD-7)	**0.61**	**0.29**	0.25	**0.035**
Depressive symptoms (EPDS)	**0.62**	**0.26**	**0.27**	**0.021**
Hostility (BSI)	−0.38	0.35	−**0.12**	0.274
Perceived social support (BSSS)	−**1.31**	**0.50**	−0.21	**0.010**

*B and SE B, non-standardized coefficients; β, standardized coefficient; bold font indicates statistical significance; GAD-7, seven-item Generalized Anxiety Disorder Scale; EPDS, Edinburgh Postnatal Depression Scale; BSI, Brief Symptom Inventory; BSSS, Berlin Social Support Scale*.

For overall pregnancy-related worries, a total of 28.2% of variance (*R*^2^_adj_ = 0.28, *p* = 0.000) was explained by the included variables. After controlling for gestational age at assessment, significant associations with household income, symptoms of generalized anxiety, depressive symptoms, as well as perceived social support were found. The associations with paternal age and hostility were no longer significant. Thus, fathers with lower income, higher symptom levels of anxiety and depression, and lower perceived social support reported higher levels of pregnancy-related worries.

### Sensitivity Analyses

Regression analysis was repeated (a) with backwards entry of the data and (b) based on cases with full data sets only, which confirmed stability of results.

### Supplementary Analyses

Previous research in mothers not only identified specific domains of pregnancy-related worries but also indicated domain-specific associations with relevant factors (42, 51, 104, 128). Thus, to investigate associations of the specific domains of pregnancy-related worries with the included variables, individual regression analyses were conducted with the CWS subscales *socioeconomic and relationships* (housing, money problems, employment, relationships with partner or family and friends), *socio-medical* (going to hospital, internal examinations, giving birth, coping with the new baby), and *health of the baby* (something wrong with the baby, possibility of miscarriage) as outcome variables (see Supplementary Material for detailed results on regression analyses). Reliability was satisfying and acceptable for the subscales *socio-medical* (α = 0.77) and *health of the baby* (α = 0.78). For the *socioeconomic and relationships* scale, reliability was below satisfying (α = 0.66). Since reliability was for *health of oneself/someone close* far below acceptable (α = 0.30), this subscale was excluded from further analyses.

For the CWS subscale *socioeconomic and relationships* (*M* = 1.06, *SD* = 0.71), 35.2% of total variance (*R*^2^_adj_ = 0.352, *p* = 0.000) was explained by the included variables. After controlling for gestational age at assessment, household income, generalized anxiety, and perceived social support remained significantly associated with this CWS subscale. Fathers with lower income, higher generalized anxiety levels, and lower perceived social support reported higher levels of prenatal concerns in the context of socioeconomic aspects and relationships.

For the CWS subscale *socio-medical* (*M* = 0.97, *SD* = 0.79), 11.9% of total variance (*R*^2^_adj_ = 0.119, *p* = 0.001) was explained by the included variables. Only having previous children was significantly associated with socio-medical concerns. Thus, expecting the first child was associated with higher socio-medical concerns.

For the CWS subscale *health of the baby* (*M* = 1.36, *SD* = 1.08), 14.8% of variance (*R*^2^_adj_ = 0.148, *p* = 0.001) was explained by the included variables. After controlling for gestational age at assessment, paternal age and depressive symptoms significantly explained variance in the outcome. Thus, fathers whose partners had not given birth before, younger fathers, and those with higher levels of depressive symptoms reported higher levels of concerns related to the health of the baby.

## Discussion

The first aim of this study was to investigate the relevance of a broad range of worries related to pregnancy and becoming a parent from the prenatal paternal perspective. Results in this sample from the general population show that overall worries in expectant fathers were rather low. The strongest concerns centered around health-related but also socioeconomic issues that are likely to be affected by parenthood. The second aim was to investigate associations of pregnancy-related worries with socioeconomic, obstetric, as well as psychosocial variables.

### Distribution of Concerns

On item level, expectant fathers with major worries regarding specific prenatal aspects could be identified. 10.8% of fathers reported major worries related to the health of someone close to them, and 10.1% expressed major concerns related to something being wrong with the baby, which is in line with previous studies (34). Only few men reported major worries related to childbirth itself (3.1%). Internal examinations and going to the hospital were no major concerns. In previous research on childbirth-related concerns, percentages of highly anxious fathers ranged from 10.9 to 54.3% (33, 35, 63). However, due to methodological differences in the assessment of fear of childbirth as a distinct concept, our results are not directly comparable to these studies (33, 35, 65). Overall, men reported low levels of concern, also in comparison to Lindgren et al.'s (62) study, where fathers scored lower on socio-medical and health-related subscales of the CWS than mothers, and relationships were the lowest ranked concerns in both mothers and fathers. Socioeconomic aspects were however not investigated in the context of their study. In the current sample, socioeconomic concerns regarding the financial and employment situation were among the stronger paternal concerns, in line with results by Forsyth et al. (72) in a sample from Australia and also by Biehle and Mickelson (73) in fathers from the USA. In a German study investigating pregnancy-related worries with the CWS in a sample of 344 pregnant women from the general population (76), concerns related to financial and employment problems were less intense than in our paternal sample and women scored highest in concerns centering around childbirth itself and something being wrong with the baby. In many families in Germany, both partners contribute to the household income. In families with underaged children, 74.7% of mothers and 92.9% of fathers were employed in 2019 (129). It is also common in Germany that mothers are on maternity leave from the last 6 weeks before childbirth and often up to at least 1 year after the child is born (130, 131). Even though mothers are provided with financial state support, during that time, the father's employment is often the main source for the family income. This might lead to higher pressure in fathers and be reflected in the stronger concerns related to financial aspects compared to the maternal sample of Petersen et al. (76). However, in another German study, not being able to provide financially for the child was among the lower concerns not only in mothers but also in fathers (74). One explanation for these divergent results could be that not having enough financial resources to cover specific expenses related to childcare might not be a relevant cause for concern, but the overall financial situation due to these expanses along with a reduced household income due to pregnancy and maternity leave might be. From an international perspective, the general relevance of specific concerns might greatly vary depending also on family-friendly politics. For example, families in Sweden or Germany are entitled for employment-protected, paid maternity leave and home care leave for mothers or fathers (132). However, comparing for example the OECD countries, some do not offer financial support after giving birth, or, as in the USA, neither maternity nor home care leave. Thus, parental concerns related to the socioeconomic situation might be even stronger in those countries with little or no financial support from the government. Previous literature supports this assumption: While, in the current sample, <10% reported major concerns related to finances and the employment situation, 30% of Biehle and Mickelsons (73) US sample and 52% of Forsyth et al. (72) Australian sample reported financial concerns. Noteworthy, samples in these studies reported an average to high socioeconomic background and samples were rather small. Therefore, international research in larger, heterogeneous samples is clearly needed to further investigate the relevance of these concerns for expectant fathers with diverse socioeconomic and cultural backgrounds and living conditions.

### Associations With the Included Variables

Regarding the association with contextual factors, a negative bivariate correlation with paternal age showed that younger fathers reported higher pregnancy-related worries, which is in line with previous literature (63, 74). However, in multiple regression analysis, paternal age did not significantly explain variance in pregnancy-related worries. This might be caused by a positive association of paternal age with household income, with older fathers reporting a significantly higher household income than younger fathers. In multiple regression analysis, household income on the other hand significantly explained variance in pregnancy-related concerns, with higher income being associated with lower pregnancy-related worries. Therefore, in this sample, the association of paternal age with pregnancy-related worries might be an indirect one, mediated by household income. The negative association with household income is further in line with results in studies on general anxiety in men (3) and pregnancy-related worries in women (49, 85). Supplementary analyses showed that associations were significant with the socioeconomic and relationships subscale. Thus, fathers with lower income were rather concerned about socioeconomic aspects than medical procedures, birth, or the health of the baby. This could be explained by the comparison to many other countries' well-developed perinatal health care, often covered by health insurance. Thus, low-income parents in Germany might receive the same level of basic standard care as those with higher socioeconomic status, potentially helping them to reduce concerns. Globally, the availability and accessibility of, for example, early standardized antenatal care vary substantially on the regional level and between income groups (133). Further, evidence from middle- and low-income countries indicates an association of fewer antenatal care visits with maternal education and income, being a single parent, living in rural areas, and higher household size and parity (134). On the background of reported associations of antenatal classes and education with parental pregnancy-related concerns, preparation for parenthood, and mental health (29, 63, 135), less opportunities for antenatal care or education classes might also lead to higher paternal insecurities and concerns.

On an overall level, pregnancy-related worries were, against our hypothesis, not significantly associated with having previous children or miscarriages as reported by the partner. However, as indicated in the supplementary analyses of the subscales, having previous children was the only factor significantly explaining variance in socio-medical worries. In line with previous research (35, 63), those fathers in the current analysis whose partners had given birth before were less concerned about medical procedures, childbirth, going to the hospital, or coping with the new baby, which might be due to first experiences with delivery. Regarding miscarriages, our results are not in line with previous qualitative and quantitative research indicating higher pregnancy-related concerns also in fathers after perinatal loss (94–96). Still, the level of worry regarding a miscarriage in the participating fathers was comparable or even slightly higher than in a sample of German pregnant women (76). In contrast to earlier studies, on the bivariate level, a non-significant trend was observed, indicating lower worries in men whose partner reported a previous miscarriage. A positive correlation between miscarriages and number of children might partially explain the unexpected direction of effects between miscarriages and pregnancy-related worries, in a way that many of the women with the experience of prenatal loss of a pregnancy might potentially also have had at least one successful pregnancy as well. Also, in the current study, pregnancy-related worries were assessed in the second to third trimester when the highest risk of having a miscarriage is usually overcome. Perinatal care in Germany includes several routine screenings starting early in pregnancy (136), which might also help to reduce worries about potential miscarriages. Future research should investigate these associations and the unexpected trend observed in the current study.

Regarding psychosocial variables, pregnancy-related worries were higher in men reporting also higher levels of anxiety and depression, which is in line with previous research (59). In the supplementary analyses, symptoms of generalized anxiety were only associated with socioeconomic and relationship aspects. One explanation for this result might be that concerns related to the family's financial situation and to the partner or social relationships might feel less controllable. Also, higher depressive symptoms were associated with worries related to the health of the fetus or the possibility of a miscarriage. For fathers, the lack of a physical connection to the baby, including also the possibility to directly feel changes in, for example, movement of the child, might lead to the perception that the health of the baby is beyond their control, eventually increasing the risk for catastrophizing. Depression and anxiety with their negative basic assumptions and a tendency to catastrophize have been associated with more worry, also regarding pregnancy-related topics (42, 104). Further, the size of the associations reported here indicate in line with research in maternal samples that pregnancy-related worries are related to but still distinct from general levels of anxiety and depression (42, 51, 128), which underlines the importance of further investigating pregnancy-specific concerns in fathers as a distinct entity.

Since previous research indicates that men might tend to express anxious-depressive mood more often in externalizing behavior [e.g., (107)], hostility was included as a potential associated factor. Despite a positive bivariate correlation, hostility did not significantly explain variance in pregnancy-related concerns in multivariate analysis. This might be due to a higher overlap of anxious and depressive symptoms with pregnancy-related concerns. Another explanation might be the rather small variance in hostility scores. Even though comparable to the scores from the general population (118), the mean hostility scores were rather low. Due to the proposed potential gender-related differences in manifestation of prenatal distress, it might be of interest to further investigate these associations in clinical samples.

Finally, perceived social support was associated with pregnancy-related worries, which is in line with previous results indicating the protective effect of social support on perinatal mental health and specifically pregnancy-related worries (59, 108). The pregnant partner as well as family and friends are generally seen by fathers as an important source of pregnancy-related information (72). Thus, having a strong supportive social network with peers, family, or friends that can additionally give advice, potentially also based on their own experiences, might help to buffer insecurities arising during the perinatal period.

The results of this study support the assumption that a wider assessment of pregnancy-related and general worries (75) is valuable to describe the perspective of expectant fathers in the prenatal period sufficiently. Still, there are some methodological issues that should be considered in future research when using the CWS with expectant fathers. The wording of the item “the health of someone close to you” did not clearly differentiate between worry regarding the health of the pregnant partner or another close person. Even though it is likely that in the context of this study fathers refer to their pregnant partner when answering this question, the item leaves room for interpretation. Further, an investigation of the psychometric properties of the German version of the CWS (76) and its subscales in expectant fathers is still needed. Thus, and due to the partly low scale reliability scores, especially for the subscales *socioeconomic and relationships* and *health of the baby/other*, the results of the supplementary analyses should be interpreted with caution. While questionnaires assessing fear of childbirth have been used in fathers more often in the past (34), questionnaires have been individually developed for specific study purposes (74) or available questionnaires have been adapted to the fathers' needs (122); validated questionnaires assessing paternal concerns beyond this scope are still missing and it is possible that not all aspects relevant for fathers are covered by the existing literature. For example, in Greer's (66) study, the interviewed fathers had the strongest concern regarding the consequences of a difficult birth on the mental health of their partner. Further concerns that could especially arise in fathers during the prenatal period are related to finding their own role and being able to properly support their pregnant partner during birth [e.g., (87)]. To identify men suffering from pregnancy-related worries already in prenatal care, specific instruments need to be available and evaluated in expectant fathers (59).

### Limitations

This population-based sample was rather homogeneous in terms of sociodemographic background. All participants were in a relationship with their pregnant partner. Another limitation is that the questionnaire-based assessment required a good understanding of the German language. These characteristics might limit generalizability of the results. Additionally, a selection bias might be possible, since only interested fathers who accompanied their partner to one of the study appointments were recruited. Further, the current analysis is based on a cross-sectional assessment, which can highlight associations among variables but does not allow any causal interpretation of effects. Due to methodological reasons, it was not possible to include current pregnancy complications as a predictor variable. Parity and previous miscarriages were reported by the pregnant partner and not assessed individually for the expectant father. It is thus possible that the experience with birth or miscarriages might differ between the assessed men and their partners. Further, due to excluding women being pregnant by reproductive technologies, women with a history of infertility might not be adequately represented in this study. It would be of interest to replicate our findings in a larger, heterogeneous sample including participants with high-risk status regarding their socioeconomic or psychosocial background and obstetric history.

### Implications

The results of this study give further insight into pregnancy-specific concerns in a German sample from the general population. They indicate that pregnancy-related worries were overall rather low in expectant fathers in our study, who had an overall average to high educational and income level and were living in a relationship with the mother of their child and in a Western country with a universal health care system and coverage of antenatal care independent of income or education, as well as entitlement to paid maternity leave and parental home care. However, for some individual aspects, the men in the current study reported levels of major worry. Our results on associated factors and previous research indicate that younger first-time fathers with higher levels of anxiety or depression, fewer financial resources, and less social support should get more attention in the context of prenatal care.

Still, fathers express that their perspective and particularly their emotional well-being are often not recognized in prenatal care or they hesitate to express these concerns themselves. Addressing prenatal worries in both parents actively already prenatally, during ultrasound, or as part of birth preparation classes might make expectant fathers feel more involved. Moreover, father-specific settings for men in prenatal routine, similar to baby massage classes for fathers in the postpartum period, could serve as a peer-to-peer forum to simultaneously share potential paternal worries or fears and promote additional social support (36). This is not only important for the well-being of the father but might also foster paternal involvement in caretaking from the beginning with its positive consequences for the developing father–child relationship. Besides birth-related topics, more general pregnancy-related issues that may also arise in the transition to parenthood such as socioeconomic concerns, as well as the role of being the supportive partner during birth, should be addressed and investigated in future research. Most studies investigating pregnancy-related worries in fathers stem from European and especially Scandinavian countries. However, the relevance of specific anxieties and concerns might vary depending on the general access to prenatal care in the specific region or country as well as on the socioeconomic situation of the individual family. Moreover, different cultural backgrounds might substantially affect the societal and personal expectations on the paternal role and its potentially associated worries. To enable a comparison across countries and cultures regarding the generalizability of results, qualitative and quantitative analyses in samples from diverse backgrounds are needed to identify not only relevant concerns and worries for expectant fathers but also associated factors related to specific domains of pregnancy-related worries. For a general understanding, longitudinal studies are needed to investigate not only the development of pregnancy-related worries during pregnancy but also their relevance for the postnatal period.

## Data Availability Statement

The datasets presented in this article are not readily available because of the ethical committee's decision. Requests to access the datasets should be directed to a.goebel@uke.de.

## Ethics Statement

The studies involving human participants were reviewed and approved by Ethics Committee of the Hamburg Chamber of Physicians(application numbers PV3694, PV5574), Hamburg Chamber of Physicians, Hamburg, Germany. The patients/participants provided their written informed consent to participate in this study.

## Author Contributions

All authors contributed substantially to this study. AG developed the research question, conducted the statistical analyses, drafted and revised the manuscript. PA and AD contributed to the final research question and the research design, the drafting and revision of the manuscript. KH and MS-M contributed to the development of the final research question and the research design, the drafting and revision of the manuscript. SM developed the research question, contributed to the research design, drafted and revised the manuscript. All authors approved the final version of this manuscript.

## Conflict of Interest

The authors declare that the research was conducted in the absence of any commercial or financial relationships that could be construed as a potential conflict of interest.
