# Supplementary material for: Manifestation and Associated Factors of Pregnancy-Related Worries in Expectant Fathers
Source: Front Psychiatry. 2020 Dec 11;11:575845. doi: 10.3389/fpsyt.2020.575845 (PMC7759496; doi:10.3389/fpsyt.2020.575845)
Supplement: Supplementary file 1 [file Table_1.doc]

**Supplementary Table**

| **S1.** Prediction of specific pregnancy-related worries | | | | | | | | | | | | | | | | | | | |
| --- | --- | --- | --- | --- | --- | --- | --- | --- | --- | --- | --- | --- | --- | --- | --- | --- | --- | --- | --- |
|  | | Socio-economic and relationships | | | | |  | | Socio-medical | | | | | Health of the baby | | | | | |
| Variable | | *B* | SE *B* | β | *p* |  | | *B* | | SE *B* | β | *p* | *B* | | | SE *B* | β | | *p* |
|  | Constant | 4.51 | 0.82 |  | 0.000 |  | | 3.64 | | 1.06 |  | 0.001 |  | | 6.03 | 1.54 |  | 0.000 | |
|  | Gestational age | -0.00 | 0.01 | -0.04 | 0.584 |  | | -0.00 | | 0.01 | -0.03 | 0.721 |  | | **-0.02** | **0.01** | **-0.20** | **0.022** | |
|  | Paternal age | -0.02 | 0.01 | -0.13 | 0.107 |  | | -0.02 | | 0.02 | -0.10 | 0.293 |  | | **-0.50** | **0.02** | **-0.20** | **0.024** | |
|  | Household income | **-0.11** | **0.03** | **-0.28** | **0.000** |  | | -0.07 | | 0.04 | -0.17 | 0.060 |  | | -0.06 | 0.06 | -0.10 | 0.251 | |
|  | Previous children (no/yes) | -0.02 | 0.11 | 0.00 | 0.986 |  | | **-0.40** | | **0.15** | **-0.25** | **0.008** |  | | -0.35 | 0.20 | -0.16 | 0.085 | |
|  | Miscarriages (no/yes) | -0.01 | 0.15 | -0.01 | 0.931 |  | | -0.11 | | 0.19 | -0.05 | 0.486 |  | | 0.43 | 0.26 | 0.15 | 0.106 | |
|  | Generalized anxiety (GAD-7) | **0.08** | **0.02** | **0.41** | **0.000** |  | | 0.02 | | 0.03 | 0.09 | 0.486 |  | | 0.00 | 0.04 | 0.00 | 0.958 | |
|  | Depressive symptoms (EPDS) | 0.02 | 0.02 | 0.13 | 0.221 |  | | 0.04 | | 0.03 | 0.17 | 0.161 |  | | **0.78** | **0.04** | **0.27** | **0.027** | |
|  | Hostility (BSI) | -0.02 | 0.03 | -0.07 | 0.485 |  | | -0.02 | | 0.04 | -0.06 | 0.631 |  | | 0.01 | 0.05 | 0.02 | 0.875 | |
|  | Perceived social support (BSSS) | **-0.13** | **0.04** | **-0.27** | **0.001** |  | | -0.89 | | 0.05 | -0.15 | 0.087 |  | | -0.13 | 0.07 | -0.17 | 0.061 | |
|  | *B* and SE *B* nonstandardized coefficients, β standardized coefficient; bold font indicates statistical significance; GAD-7= seven-item Generalized Anxiety Disorder Scale; EPDS = Edinburgh Postnatal Depression Scale; BSI = Brief Symptom Inventory; BSSS = Berlin Social Support Scale | | | | | | | | | | | | | | | | | | |
